# Morphology control of organic halide perovskites by adding BiFeO_3_ nanostructures for efficient solar cell

**DOI:** 10.1038/s41598-019-51273-y

**Published:** 2019-10-28

**Authors:** Haowen Xu, Heyi Zhang, Yuhui Ma, Mao Jiang, Yewei Zhang, Yinan Wu, Haoran Zhang, Ruidong Xia, Qiaoli Niu, Xing’ao Li, Wei Huang

**Affiliations:** 1 Key Laboratory for Organic Electronics and Information Displays & Institute of Advanced Materials, Jiangsu National Synergetic Innovation Center for Advanced Materials (SICAM), Nanjing University of Posts and Telecommunications, 9 Wenyuan Road, Nanjing, 210023 P.R. China; 2 New Energy Technology Engineering Laboratory of Jiangsu Provence & School of Science, Nanjing University of Posts and Telecommunications (NUPT), Nanjing, 210023 P.R. China; 3 Shanxi Institute of Flexible Electronics (SIFE), Northwestern Polytechnical University (NPU), 127 West Youyi Road, Xian, 710072 P.R. China; 4 Institute of Materials Science and Engineering, Nanjing University of Posts and Telecommunications, 9 Wenyuan Road, Nanjing, 210023 P.R. China

**Keywords:** Devices for energy harvesting, Solar cells

## Abstract

The morphology of perovskite light-absorption layer plays an important role in the performance of perovskite solar cells (PSCs). In this work, BiFeO_3_ (BFO) nanostructures were used as additive for CH_3_NH_3_PbI_3_ (MAPbI_3_) via anti-solvent method. The addition of BFO nanostructures greatly enhanced the crystallinity, grain size and film uniformity of MAPbI_3_. As a result, the charge carrier mobility and electron diffusion length increased, leading to the increase of the short circuit current (J_SC_) of PSCs. This work provides a very simple but effective approach to improve the morphology of perovskite layer for efficient PSCs.

## Introduction

Organic-inorganic halide perovskite solar cells (PSCs) have become the future photovoltaic technology due to their low-cost fabrication technology and high power conversion efficiency (PCE), which can be ascribed to the unique properties of organic-inorganic halide perovskites, such as high carrier mobility, high absorption coefficients, long charge carrier diffusion length and small exciton binding energy^1,2^. In particular, MAPbI_3_ was the first widely used perovskite material in PSCs^3,4^. During the past nine years, many approaches were studied to promote the performances of MAPbI_3_ based PSCs, such as, elements doping^5,6^, solvent engineering^7,8^ and thermal treatment^9^. Remarkably, the PCE of PSCs were boosted from 3.8% to 23.2%^10^.

Despite these achievements, several challenges still remain. One of the greatest challenges is to obtain pinhole-free perovskite layer with large grains by using simple techniques^11–13^. Defects and grain boundaries within the perovskite layer can trap charge carriers, leading to the recombination of electrons and holes, and therefore decrease the PCE of PSCs. While, perovskite layer with larger grains contains less grain boundaries, which will facilitate the transport and collection of charge carriers^14,15^. Thus, the obtaining of high-quality perovskite film with large and uniform grains is of critical importance to achieve highly efficient PSCs.

To date, many methods have been selected to improve the morphology of organic-inorganic halide perovskite, such as using perovskite-fullerene bulk heterojunction structure^16,17^, optimizing the perovskite precursor solution^18^, thermal annealing^19,20^, intramolecular exchange^21,22^ and solvent annealing^23,24^. In addition, additive engineering is another effective method^25–28^. For example, by adding a fullerene derivative (α-bis-PCBM) via anti-solvent method, the vacancies and grain boundaries of the perovskite film was filled, leading to the enhanced crystallization of perovskite^27^; sulfonate carbon nanotubes (s-CNT) incorporated perovskite film had large grain size and filled grain boundary, which directly improved the device performance^28^. Although the additive engineering is effective, however, only limited additives were reported.

Here, BFO nanostructures were used as an additive for MAPbI_3_, the active layer of PSCs, via anti-solvent method. Interestingly, BFO has the similar perovskite structure with organic-inorganic halide perovskites, such as MAPbI_3_. Experimental results showed that the addition of BFO nanostructures increased the grain size and crystallinity of MAPbI_3_, which enhanced the charge carrier mobility and electron diffusion length. As a result, the performances of PSCs were enhanced. The influence of BFO’s morphology on the quality of MAPbI_3_ and performance of PSCs was also studied. It was found that both nanosheets BFO (s-BFO) and nanocuboids BFO (c-BFO) effectively improved the morphology of MAPbI_3_, leading to the enhancements of PCE values of PSCs from 13.8 ± 0.82% of the control device to 15.08 ± 0.56% and 15.81 ± 0.52%, respectively. Our work expands the type of additives to improve the morphology of MAPbI_3_ and the performance of PSCs.

## Results

**Figure 1:**
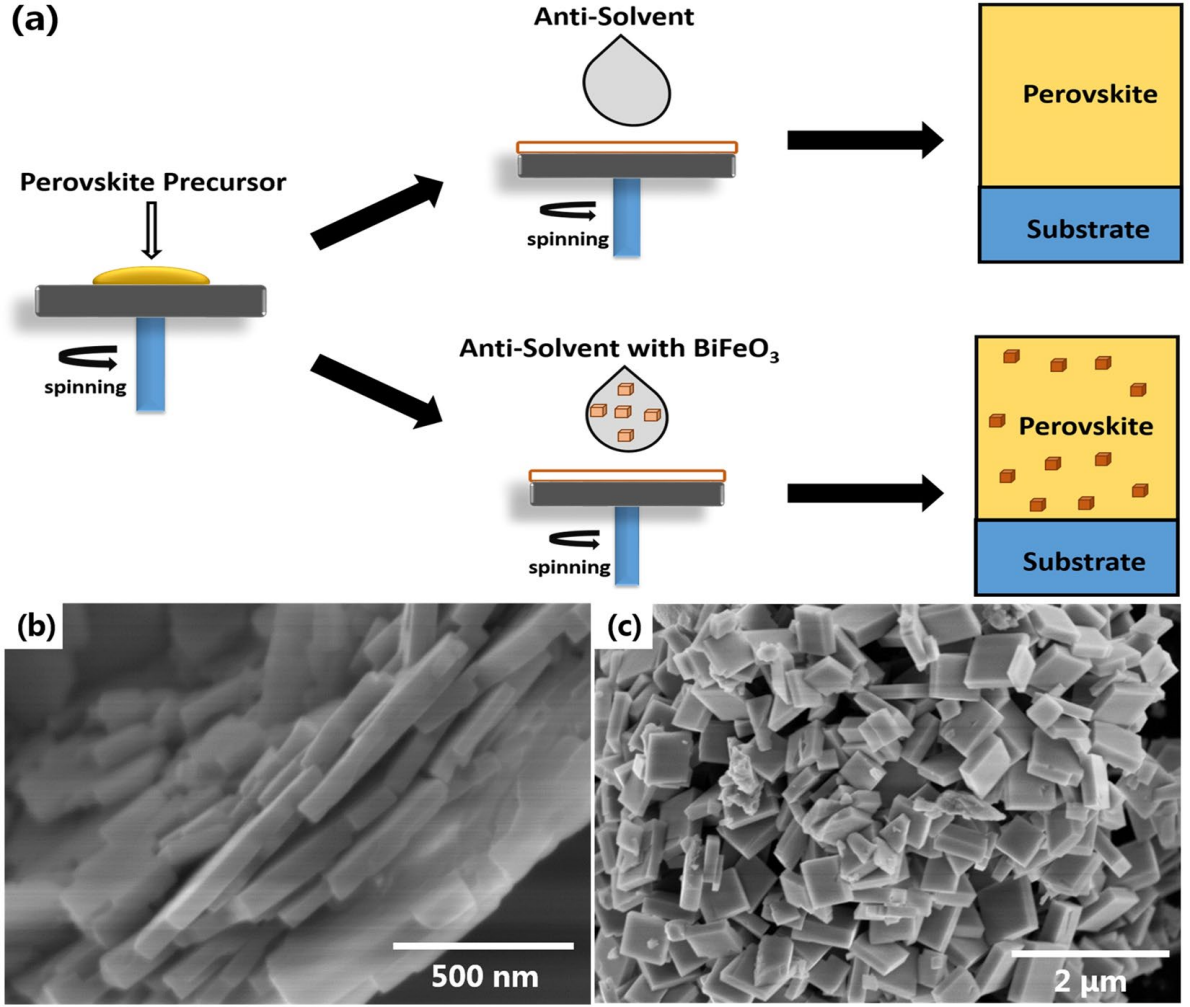
(**a**) The detailed process procedures for the preparation of MAPbI_3_, the SEM images of s-BFO (**b**) and c-BFO (**c**).

One-step solution method was used to fabricate MAPbI_3_ film, as depicted in Fig. 1(a). During the spin-coating of MAPbI_3_ precursor solution, anti-solvent toluene with BFO nanostructures was dripped. Pure toluene without BFO nanostructures was used to fabricate the pristine perovskite film and control device. Two kinds of BFO nanostructures, nanosheets (s-BFO) and nanocuboids (c-BFO), were studied, as shown in Fig. 1(b,c). The s-BFO is flake-shaped with a thickness of about 60 nm. The c-BFO is cuboid-shaped with a thickness of about 90 nm, and both the length and width of about 300 nm.

**Figure 2:**
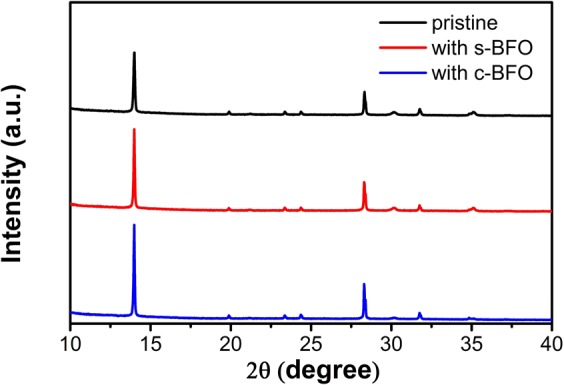
XRD patterns of MAPbI_3_ films with or without BFO.

The crystal structures of pristine MAPbI_3_ films and MAPbI_3_ films with s-BFO or c-BFO were characterized by XRD, as shown in Fig. 2. All MAPbI_3_ films have two strong diffraction peaks at 14.1° and 28.4°, corresponding to the (110) and (220) planes of MAPbI_3_, respectively^29^. With the addition of BFO, no new diffraction peaks were observed. It indicated that the addition of BFO didn’t change the orthorhombic crystal structure of MAPbI_3_. While, with the addition of BFO, the intensity of diffraction peaks increased, together with the decrease of full width at half maximum (FWHM) values. For the strongest peak at 14.1°, the peak intensity increased from 14343 of the pristine film to 18867 and 20557 of the films with s-BFO and c-BFO, and the FWHM values decreased from 0.081° to 0.079° and 0.077°, respectively. It indicated the obviously increased crystallinity of MAPbI_3_ by adding BFO nanostructures^30^.

**Figure 3:**
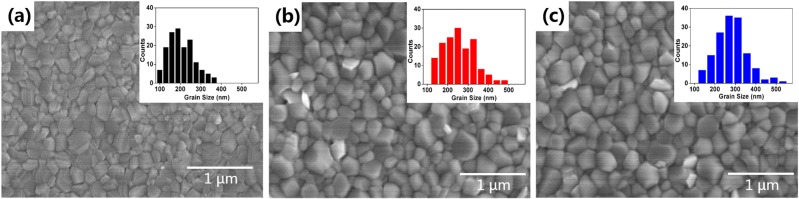
SEM images of MAPbI_3_ films: (**a**) pristine, (**b**) with s-BFO and (**c**) with c-BFO. Insets of the images are the statistics of crystal sizes.

To study the morphology change of MAPbI_3_, SEM images were taken, as shown in Fig. 3. For all MAPbI_3_ films, densely arranged grains fully covered the substrate. It is notable that the addition of BFO increased the crystal size of MAPbI_3_. Insets of the SEM images in Fig. 3 are the statistics of crystal sizes. The average crystal size is 181 nm for the pristine film, which increased to 265 nm (with s-BFO) and 281 nm (with c-BFO). In addition, Fig. 3 shows that the crystal size distribution of MAPbI_3_ film with c-BFO is more centralized than the s-BFO added film, indicating more uniform crystals in size. Perovskite layer with uniform crystals facilitates the transportation of charge carriers^23^.

Surface roughness is one of the key issues affecting the performance of PSCs. Therefore, atomic force microscope (AFM) images of MAPbI_3_ films were measured as presented in Figure S1. The root mean square (rms) roughness of MAPbI_3_ films with s-BFO and c-BFO were 7.98 nm and 6.54 nm, respectively, which were smaller than 8.73 nm of the pristine film. MAPbI_3_ film with smoother surface has less defects, which is good for the charge separation and transportation at the interface of MAPbI_3_ and [6,6]-phenyl-C61-butyric acid methyl ester (PCBM)^31^.

The positive effect of BFO to the morphology of MAPbI_3_ film was confirmed from the above analysis. To understand the mechanism of BFO treatment, it is necessary to examine the residual of BFO in MAPbI_3_ film. Energy dispersive spectroscopy (EDS) analysis is an effective method to examine the distribution of elements in thin films^32,33^. We performed EDS on MAPbI_3_ films, the spectra and elemental mapping were shown in Figure S2. During the EDS measurements, the detected elements were auto marked. The EDS spectra and elemental mapping data were shown as received without any further processing. We can see that for the MAPbI_3_ film with BFO, Bi and Fe were detected except for the common elements such as Sn, In and Al from ITO or glass substrate. However, XRD patterns (Fig. 2) indicated that BFO didn’t exist in the crystal lattice of MAPbI_3_. Therefore, we can conclude that BFO participates in the growth of MAPbI_3_ crystals without altering the crystal structure of the MAPbI_3_. The addition of BFO enhanced the crystallinity and grain size of MAPbI_3_ film and reduced its surface roughness. These observations suggest that the added BFO nanostructures during the spin-coating of perovskite precursor solution induced the nucleation of perovskite crystals^34,35^, which promoted the crystal growth of MAPbI_3_. Compared with s-BFO, grainy c-BFO was facilitate to the homogeneous growth of MAPbI_3_ crystals, resulting in more uniform MAPbI_3_ crystals in size. MAPbI_3_ film with larger grains have less defects, lower density of grain boundaries and a smoother surface.

**Figure 4:**
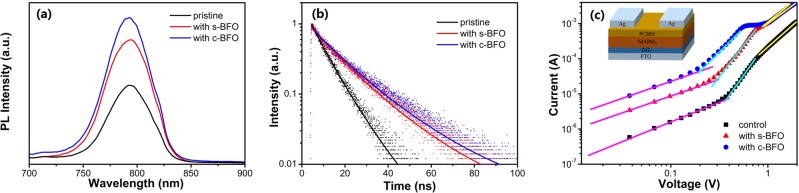
(**a**) Photoluminescence (PL), (**b**) time-resolved PL (TRPL) spectra of MAPbI_3_ films with or without BFO, and (**c**) dark I-V curves of the electron-only devices. The inset shows the structure of the electron-only device.

To study the charge carrier dynamics, photoluminescence (PL) and time-resolved PL (TRPL) spectra of MAPbI_3_ films on quartz substrate were measured as shown in Fig. 4(a,b). With the addition of BFO, the PL intensity increased, indicating the decrease of defects in the MAPbI_3_ film^36^. Meanwhile, the TRPL measurement demonstrated an increased charge carrier lifetime from 7.95 ns for the pristine MAPbI_3_ film to 17.10 ns and 17.81 ns for the films with s-BFO and c-BFO, respectively. It suggested the reduction of recombination in MAPbI_3_, which can be attribute to the crystal size increase and surface roughness decrease. They were in favor of the transporting of electrons and holes^25^. Therefore, the J_SC_ of PSCs could be improved.

Electron-only devices with the structure of ITO/TiO_2_/MAPbI_3_/PCBM/Ag were fabricated to evaluate the trap density and electron mobility of MAPbI_3_. Figure 4(c) shows the current-voltage (I-V) curves of the electron-only devices under dark. The linear correlation at low bias voltage (magenta line) reveals an ohmic-type response. The current increased rapidly (cyan line) when the bias voltage is higher than the kink point, which is defined as the trap-filled limit voltage (V_TFL_), indicating that the traps are completely filled. The trap density (N_t(e)_) can be obtained by using Eq. (1)^37^.

1 $${N}_{t(e)}=\frac{2{\rm{\varepsilon }}{{\rm{\varepsilon }}}_{0}{V}_{TFL}}{e{L}^{2}}$$

Where ε is the relative dielectric constant^38^, ε_0_ is the absolute dielectric constant, L is the thickness of perovskite film, and e is the elementary charge. When the voltage increases to a certain level, the increase of current slows down with the increase of voltage. The relationship between current and voltage conforms to space-charge-limited current model (SCLC), from which the carrier mobility (μ_e_) can be estimated by the Mottley-Gurney’s law^39^. Furthermore, the diffusion length of electron can be derived therefrom. The detailed values are shown in Table 1. Compared with control device, the N_t(e)_ decreased significantly from 1.579 × 10^16^ cm^−3^ to 1.255 × 10^16^ cm^−3^ and 8.506 × 10^15^ cm^−3^ for the device with s-BFO and c-BFO, respectively. The μ_e_ and diffusion length also substantially increased.

**Table 1:** The detailed values of electron trap density and charge carrier mobility of MAPbI_3_ films.

electron-only device	N_t(e)_^a^ (cm^−3^)	μ_e_^b^ (cm^2^ V^−1^ s^−1^)	diffusion length (cm)
control	1.579 × 10^16^	3.798 × 10^−5^	8.84 × 10^−8^
s-BFO	1.255 × 10^16^	2.112 × 10^−4^	3.05 × 10^−7^
c-BFO	8.506 × 10^15^	8.465 × 10^−4^	6.23 × 10^−7^

^a^N_t(e)_ is refer to trap density; ^b^μ_e_ is refer to electron mobility.

**Figure 5:**
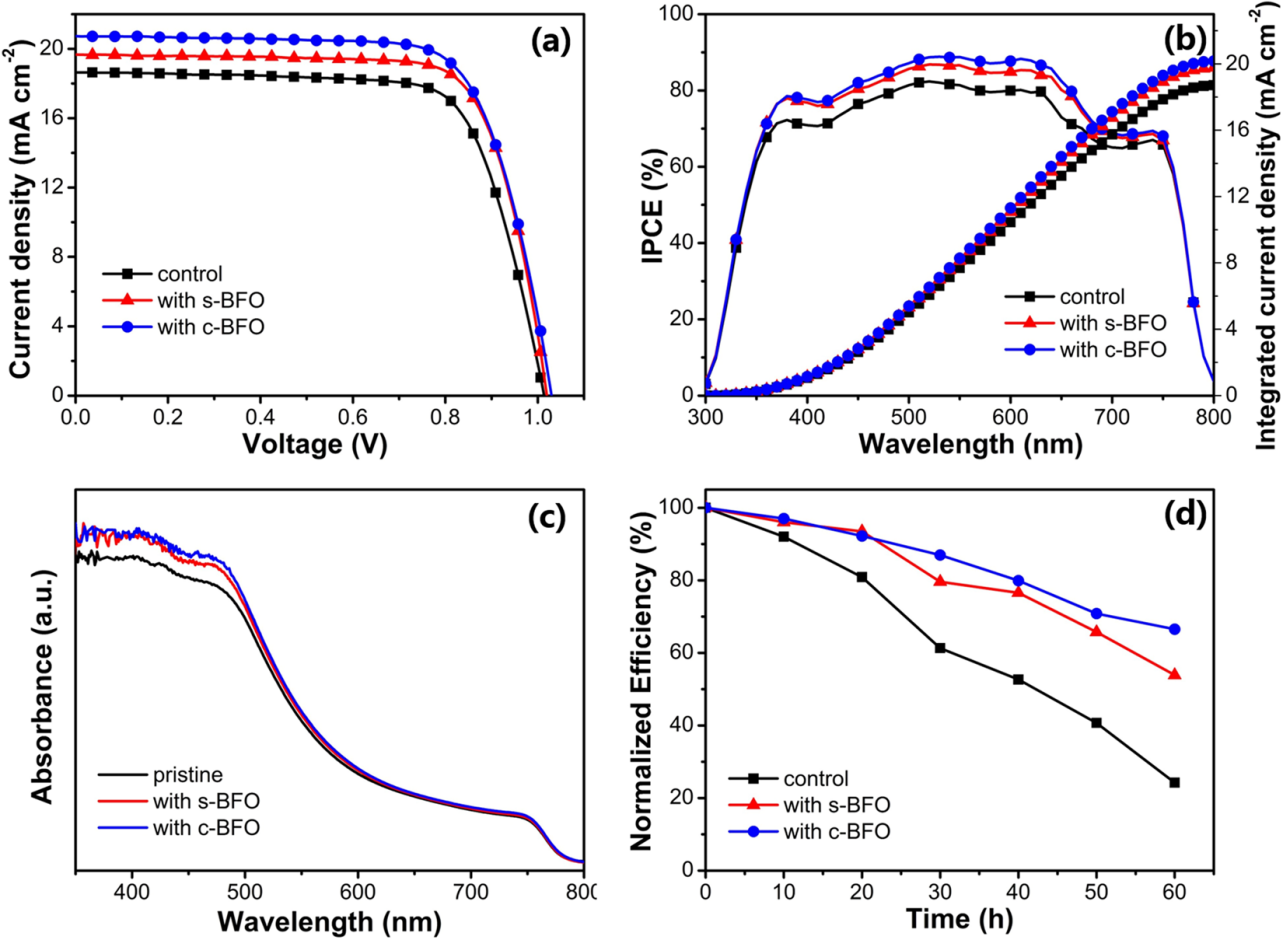
(**a**) The J-V curves, (**b**) IPCE spectra, (**c**) UV-Vis spectra of the PSCs, and (**d**) Stability test of devices.

To investigate the effect of BFO on the performances of PSCs, PSCs with device structures of ITO/NiO_x_ (20 nm)/MAPbI_3_ (290 nm)/PCBM (40 nm)/BCP (10 nm)/Ag (100 nm) were fabricated. The device configuration along with the corresponding energy band structures are depicted in Figure S3. Figure 5(a) shows the J-V curves of PSCs. The detail performance parameters of PSCs are shown in Table 2, including V_OC_, J_SC_, FF, PCE, series resistance (R_s_) and shunt resistance (R_sh_). The control device based on pristine MAPbI_3_ film has a V_OC_ of 1.01 V, J_SC_ of 18.66 ± 1.15 mA cm^−2^, FF of 73 ± 1.8% and PCE of 13.80 ± 0.82%. The values increased to 1.02 V, 19.67 ± 0.74 mA cm^−2^, 75 ± 2.2% and 15.08 ± 0.56% for PSC with s-BFO and 1.03 V, 20.74 ± 0.75 mA cm^−2^, 74 ± 1.7%, and 15.81 ± 0.52% for PSC with c-BFO. The statistics of devices performance parameters are shown in Figure S4. The results suggest that the addition of BFO mainly improved the J_SC_ of PSCs with the V_OC_ and FF almost unchanged. The enhancements of J_SC_ were further confirmed by the IPCE spectra as shown in Fig. 5(b). The integrated J_SC_ from the IPCEs were 18.7, 19.7, and 20.1, mA cm^−2^ for the control device, PSC with s-BFO and with c-BFO, respectively, which were very close to the J_SC_ obtained from the J-V scan. In addition, there was almost no hysteresis for all PSCs (Figure S5). IPCE intensity was positively related to the absorption intensity. Therefore, UV-Vis spectra on quartz substrates were studied, as shown in Fig. 5(c). MAPbI_3_ film with BFO showed stronger absorption than the pristine film in the range of 350–500 nm, which could enhance the charge carrier generation in MAPbI_3_ film. Meanwhile, although all the films have similar absorption intensity in the range of about 500 to 750 nm, the BFO added PSCs showed enhanced IPCE value. It suggested that the improvement of IPCE was also attributed to the reduced recombination loss and enhanced charge collection^40^.

**Table 2:** The detailed performance parameters of PSCs.

PSCs	V_OC_ (V)	J_SC_ (mA cm^−2^)	FF (%)	PCE (%)	R_s_ (Ω)	R_sh_ (Ω)
control	1.01	18.66 ± 1.15	73 ± 1.8	13.80 ± 0.82	85	24711
with s-BFO	1.02	19.67 ± 0.86	75 ± 2.2	15.08 ± 0.56	61	30962
with c-BFO	1.03	20.74 ± 0.92	74 ± 1.7	15.81 ± 0.52	72	34503

Stability is also a key characteristic for PSCs, which was estimated by measuring the PCE of PSCs during storing in dark at room temperature without any encapsulation (50% humidity). Figure 5(d) shows the curves of normalized PCE as a function of storage time. After 60 h storage, the PCE of BFO added PSCs maintained over 50% of their initial values, which was only 24% for the control device. The significantly improved stability of PSCs with BFO can be attributed to the larger grain size, which can effectively suppress the moisture permeation at grain boundaries^41^.

**Figure 6:**
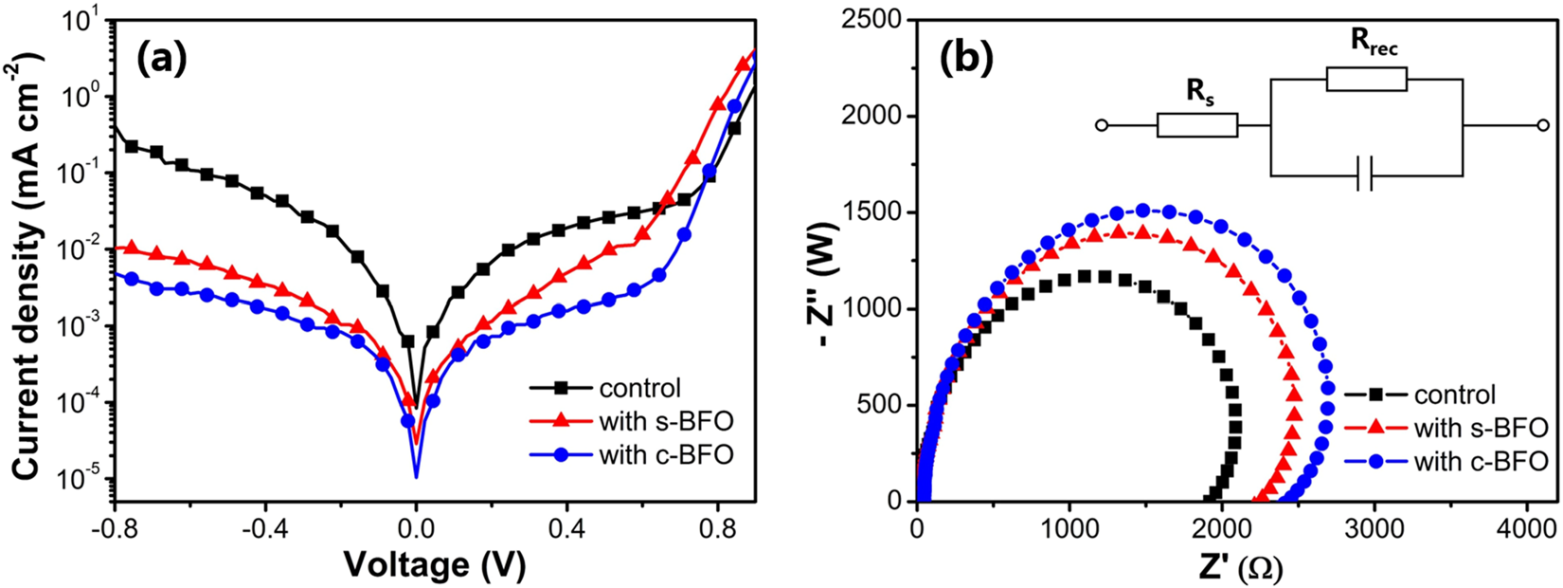
(**a**) Dark J-V curves (**b**) Nyquist plots (under dark conditions at an applied voltage of 1 V) of the PSCs, inset is the equivalent circuit.

To investigate the carrier loss and the recombination of free carriers, the dark J-V curves were collected. As shown in Fig. 6(a), with the addition of BFO, the reverse current decreased and the forward current increased, indicating the reduced leakage current and improved injection current, which can be attributed to improved morphology of MAPbI_3_ film.

The carrier transport and recombination behaviors in devices were further investigated by electrochemical impedance spectroscopy (EIS). Generally, the Nyquist plots show the combination of semicircles of the complex plane. The low frequency arc is usually attributed to the charge recombination within the perovskite film and at the interface with charge transport layer, which is often described by the recombination resistance (R_rec_). The R_rec_ for devices can be extracted by fitting the plots using the circuit shown inset of Fig. 6(b). A high R_rec_ represents less recombination and efficient dissociation of carriers at interfaces^42^. Devices with BFO shows an increased R_rec_ as compared with the control device, indicating a lower recombination rate. Therefore, the collection of charge carrier was enhanced, which was favorable for the enhancement of J_SC_ of PSCs.

## Discussion

In summary, by incorporating BFO nanostructures in anti-solvent during the spin-coating of MAPbI_3_ precursor solution, MAPbI_3_ films with increased crystallinity, larger and more uniform crystal grains were obtained. Thus, the absorption intensity of MAPbI_3_ films increased. The charge carrier mobility and diffusion length increased, and the trap density decreased. Finally, the J_SC_ of PSCs increased from 18.66 ± 1.15 mA cm^−2^ to 20.74 ± 0.92 mA cm^−2^, resulting in the PCE enhancements from 13.8 ± 0.82% to 15.81 ± 0.52%. BFO nanostructures were detected by EDS in perovskite film, which served as heterogeneous nucleation sites during the crystal growth of MAPbI_3_ film.

## Methods

### Materials and synthesis

Fe(NO_3_)·9H_2_O, Bi(NO_3_)·5H_2_O, NiCl_2_·6H_2_O, toluene and dilute nitric acid were all purchased from Sinopharm Chemical Reagent Co., Ltd. Gamma butyrolactone (GBL) and NaOH were purchased from Shanghai Macklin Biochemical Co., Ltd. The chemicals mentioned above were of analytical grade and used as received. Dimethylsulfoxide (DMSO) and chlorobenzene were purchased from Shanghai Aladdin Bio-Chem Technology Co., Ltd. Methylammonium iodide (MAI), lead iodide (PbI_2_), PC_61_BM and 2,9-dimethyl-4,7-diphenyl-1,10-phenanthroline (BCP) were purchased from Xi’an Polymer Light Technology Corp.

### Synthesis of s-BFO

1 mmol Fe(NO_3_)·9H_2_O and 1 mmol Bi(NO_3_)·5H_2_O were co-dissolved in 10 ml dilute nitric acid and 90 ml deionized water. The pH value of the solution was adjusted to 12 by adding 6 M NaOH solution. Next, the suspension was stirred for 30 min. Then, it was transferred into a 40-ml teflon-lined stainless steel autoclave and heated at 200 °C for 24 h.

### Synthesis of c-BFO

1 mmol Fe(NO_3_)·9H_2_O and 1 mmol Bi(NO_3_)·5H_2_O were co-dissolved in 10 ml dilute nitric acid and 90 ml deionized water. The pH value of the solution was adjusted to 9–10 by adding ammonia. The sediment was centrifuged out and washed with deionized water until the pH value was neutral. Then, the red sediment was re-dispersed in 4 M NaOH solution. During stirring, 2 ml PEG400 was added into the suspension. After stirring for 30 min, it was transferred into a 40-ml teflon-lined stainless steel autoclave and heated at 180 °C for 24 h.

After cooling down to room temperature, the precipitates were all centrifuged out and washed with deionized water and absolute ethanol each for three times. Finally, after drying in oven at 80 °C for 2 h, s-BFO and c-BFO were obtained.

### Preparation of NiO_x_ nanoparticle solution

12.885 g of NiCl_2_·6H_2_O was dissolved in 100 ml of deionized water under magnetic stirring. Then, 10 M NaOH solution was added into the solution drop by drop until the pH value reached 10. A turbid green solution was obtained and centrifuged. After being washed twice with deionized water, the obtained precipitation was dried at 80 °C overnight and then annealed at 270 °C for 2 h to obtain NiO_x_ nanoparticle. NiO_x_ nanoparticle solution was prepared by dispersing the NiO_x_ nanoparticles in deionized water at a concentration of 20 mg/ml. The resulted solution was filtered through a polytetrafluoroethylene (TPFE) filter (0.45 μm) before spin-coated^43^.

### Preparation of MAPbI_3_ precursor solution

MAPbI_3_ precursor solution was prepared by dissolving 1.4 mmol MAI and 1.4 mmol PbI_2_ in 1 mL GBL and DMSO (7:3/v:v). Before spin-coating, the MAPbI_3_ precursor solution was stirred at 65 °C for 12 h in N_2_ atmosphere glove box.

### Solar cell fabrication

PSCs with device configurations of ITO/NiO_x_ (20 nm)/MAPbI_3_ (290 nm)/PCBM (60 nm)/BCP (10 nm)/Ag (100 nm) were fabricated. Saturated S-BFO and c-BFO solution was prepared by dispersing S-BFO or c-BFO in toluene (0.2 mg/mL) respectively and used after stirring for 12 h and then standing for 30 min. The ITO-coated glass substrates were cleaned sequentially in detergent, deionized water, acetone, and ethanol under sonication for 20 min, respectively. After being dried by the N_2_ flow, 4 min ozone plasma at a power of 70 W was applied to remove any organic residues. Immediately, NiO_x_ nanoparticle solution was spin-coated onto the ITO glass at 4000 rpm for 30 s and then baked at 130 °C for 20 min. The substrate was transferred into a N_2_ protected glovebox with the content of water and oxygen less than 1ppm. Following, MAPbI_3_ precursor solution was spin-coated. Specifically, the spin-coating process was composed of two stages: 900 rpm for 15 s and then 4000 rpm for 25 s. For toluene washing during the spin-coating of MAPbI_3_ precursor solution, at delay time of 15 s from the beginning of the second stage, 400 μL toluene without BFO, with s-BFO or c-BFO saturated solution (0.2 mg/mL) was dripped. After being thermally annealed at 100 °C for 10 min, PCBM solution in chlorobenzene (20 mg/mL) was spin-coated at a speed of 1000 rpm for 20 s. Then, the substrates were annealed at 70 °C for 40 min. Finally, BCP and Ag were sequentially thermally evaporated at a basic pressure of 4 × 10^−4^ Pa. The active area defined by a shadow mask was 0.1 cm^2^.

### Device characterizations

The current density-voltage (J-V) characterization of PSCs was carried out using a Keithley 2400 source meter under a simulated AM 1.5 illumination (100 mW cm^−2^, NewPort, 94043 A SOLAR SIM) at a scan rate of 200 mV s^−1^. The incident-photon-to-current efficiency (IPCE) measurement was performed through an Enlitech QE-R Quantum Efficiency Measurement System (QE-R3018). The crystal structures of the MAPbI_3_ films were characterized by using a Bruker D8 ADVANCE X-ray diffraction (XRD) equipment. The morphology of the MAPbI_3_ layer was obtained using a Hitachi S-4800 field emission scanning electron microscope (SEM) and a Bruker atomic force microscope (AFM). Energy dispersive spectroscopy (EDS) was conducted by using an EDS device connected to SEM. The thicknesses of the NiO_x_, MAPbI_3_ and PC_61_BM films were determined using a Bruker DektakXT Stylus Profiler. The photoluminescence (PL) and time-resolved PL (TRPL) spectra were measured by using an Edinburgh FLS980 fluorescence spectrophotometer with an excitation at 550 nm. The impedance characteristics of the devices were measured via a Wayne Kerr 6500B analyzer.

## References

[1] Kojima A, Teshima K, Shirai Y, Miyasaka T (2009). Organometal halide perovskites as visible-light sensitizers for photovoltaic cells. Journal of the American Chemical Society.

[2] Xing G (2013). Long-range balanced electron- and hole-transport lengths in organic-inorganic CH_3_NH_3_PbI_3_. Science.

[3] Baikie T (2013). Synthesis and crystal chemistry of the hybrid perovskite (CH_3_NH_3_)PbI_3_ for solid-state sensitised solar cell applications. Journal of Materials Chemistry A.

[4] Sum TC, Mathews N (2014). Advancements in perovskite solar cells: photophysics behind the photovoltaics. Energy & Environmental Science.

[5] Zhang Y (2018). Trash into Treasure: δ‐FAPbI3 Polymorph Stabilized MAPbI3 Perovskite with Power Conversion Efficiency beyond 21%. Advanced Materials.

[6] Noh JH, Im SH, Heo JH, Mandal TN, Seok SI (2013). Chemical management for colorful, efficient, and stable inorganic-organic hybrid nanostructured solar cells. Nano Letters.

[7] Kim HB (2014). Mixed solvents for the optimization of morphology in solution-processed, inverted-type perovskite/fullerene hybrid solar cells. Nanoscale.

[8] Jeon NJ (2014). Solvent engineering for high-performance inorganic-organic hybrid perovskite solar cells. Nature Materials.

[9] Sheikh AD (2017). Effects of High Temperature and Thermal Cycling on the Performance of Perovskite Solar Cells: Acceleration of Charge Recombination and Deterioration of Charge Extraction. ACS Applied Materials & Interfaces.

[10] Jeon, N. J. *et al*. A fluorene-terminated hole-transporting material for highly efficient and stable perovskite solar cells, *Nature**Energy*, 10.1038/s41560-018-0200-6 (2018).

[11] Eperon GE, Burlakov VM, Docampo P, Goriely A, Snaith HJ (2014). Morphological Control for High Performance, Solution‐Processed Planar Heterojunction Perovskite Solar Cells. Advanced Functional Materials.

[12] Shen D (2014). Understanding the solvent-assisted crystallization mechanism inherent in efficient organic-inorganic halide perovskite solar cells. Journal of Materials Chemistry A.

[13] Hao F, Stoumpos CC, Liu Z, Chang RPH, Kanatzidis MG (2014). Controllable Perovskite Crystallization at a Gas–Solid Interface for Hole Conductor-Free Solar Cells with Steady Power Conversion Efficiency over 10%. Journal of the American Chemical Society.

[14] Nie W (2015). Solar cells. High-efficiency solution-processed perovskite solar cells with millimeter-scale grains. Science.

[15] Kim HS, Park NG (2014). Parameters Affecting I-V Hysteresis of CH_3_NH_3_PbI_3_ Perovskite Solar Cells: Effects of Perovskite Crystal Size and Mesoporous TiO_2_ Layer. Journal of Physical Chemistry Letters.

[16] Chiang, C. H. & Wu, C. G. Bulk heterojunction perovskite–PCBM solar cells with high fill factor. *Nature Photonics* **10** (2016).

[17] Wang K, Liu C, Du P, Zheng J, Gong X (2015). Bulk Heterojuntion Perovskite Hybrid Solar Cells with Large Fill-Factor. Energy & Environmental Science.

[18] Bi D (2016). Efficient luminescent solar cells based on tailored mixed-cation perovskites. Science Advances.

[19] Ye J (2017). High-temperature shaping perovskite film crystallization for solar cell fast preparation. Solar Energy Materials & Solar Cells.

[20] Yang, Y. *et al*. Enhanced Crystalline Phase Purity of CH_3_NH_3_PbI_3-x_Cl_x_ Film for High Efficiency Hysteresis-free Perovskite Solar Cells. *Acs Applied Materials & Interfaces* **9** (2017). 10.1021/acsami.7b03941 28603955

[21] Jo Y (2016). High Performance of Planar Perovskite Solar Cells Produced from PbI_2_(DMSO) and PbI_2_(NMP) Complexes by Intramolecular Exchange. Advanced Materials Interfaces.

[22] Yang WS (2015). High-performance photovoltaic perovskite layers fabricated through intramolecular exchange. Science.

[23] Xiao Z (2015). Solvent Annealing of Perovskite‐Induced Crystal Growth for Photovoltaic‐Device Efficiency Enhancement. Advanced Materials.

[24] Liu J (2015). Improved Crystallization of Perovskite Films by Optimized Solvent Annealing for High Efficiency Solar Cell. Acs Applied Materials & Interfaces.

[25] Po-Wei L (2014). Additive enhanced crystallization of solution-processed perovskite for highly efficient planar-heterojunction solar cells. Advanced Materials.

[26] Xiu G (2016). Controllable Perovskite Crystallization by Water Additive for High-Performance Solar Cells. Advanced Functional Materials.

[27] Zhang F (2017). Isomer-Pure Bis-PCBM-Assisted Crystal Engineering of Perovskite Solar Cells Showing Excellent Efficiency and Stability. Advanced Materials.

[28] Zhang Y (2016). Enhancing the grain size of organic halide perovskites by sulfonate-carbon nanotube incorporation in high performance perovskite solar cells. Chemical Communications.

[29] Zhou, Z. *et al*. Stable Inverted Planar Perovskite Solar Cells with Low‐Temperature‐Processed Hole‐Transport Bilayer. *Advanced Energy Materials* **7** (2017).

[30] Zuo C, Ding L (2015). Solution-Processed CuO and CuO as Hole Transport Materials for Efficient Perovskite Solar Cells. Small.

[31] Zuo C, Ding L (2014). An 80.11% FF record achieved for perovskite solar cells by using the NH_4_Cl additive. Nanoscale.

[32] Xie J (2017). Self‐Organized Fullerene Interfacial Layer for Efficient and Low‐Temperature Processed Planar Perovskite Solar Cells with High UV‐Light Stability. *Advanced*. Science.

[33] Liu, Z. *et al*. Efficient and stable perovskite solar cells based on high-quality CH_3_NH_3_PbI_3−x_Cl_x_ films modified by V_2_O_x_ additives. *Journal of Materials Chemistry A* 24282–24291 (2017).

[34] Bi, D. *et al*. Polymer-templated nucleation and crystal growth of perovskite films for solar cells with efficiency greater than 21%. *Nature Energy* **1** (2016).

[35] Geske T (2017). Deterministic Nucleation for Halide Perovskite Thin Films with Large and Uniform Grains. Advanced Functional Materials.

[36] Wang F (2016). Phenylalkylamine Passivation of Organolead Halide Perovskites Enabling High-Efficiency and Air-Stable Photovoltaic Cells. Advanced Materials.

[37] Dong Y (2018). High efficiency planar-type perovskite solar cells with negligible hysteresis using EDTA-complexed SnO_2_. Nature Communications.

[38] Qingfeng D (2015). Solar cells. Electron-hole diffusion lengths> 175 μm in solution-grown CH3NH3PbI3 single crystals. Science.

[39] Dong, Y. *et al*. Surface Optimization to Eliminate Hysteresis for Record Efficiency Planar Perovskite Solar Cells. *Energy & Environmental Science* **9**, 10.1039.C1036EE02139E (2016).

[40] Wu Y (2016). Perovskite solar cells with 18.21% efficiency and area over 1 cm^2^ fabricated by heterojunction engineering. Nature Energy.

[41] Chu Z (2017). Impact of grain boundaries on efficiency and stability of organic-inorganic trihalide perovskites. Nature Communications.

[42] Zhu C (2019). Strain engineering in perovskite solar cells and its impacts on carrier dynamics *Nature*. Communications.

[43] Yin X (2016). Highly Efficient Flexible Perovskite Solar Cells Using Solution-Derived NiO_x_ Hole Contacts. Acs Nano.

